# Investigating Sex Differences in Rates and Correlates of Food Addiction Status in Women and Men with PTSD

**DOI:** 10.3390/nu13061840

**Published:** 2021-05-27

**Authors:** Monika M. Stojek, Justyna Lipka, Jessica M. Maples-Keller, Sheila A. M. Rauch, Kathryn Black, Vasiliki Michopoulos, Barbara O. Rothbaum

**Affiliations:** 1 Department of Social Sciences, University of Silesia, 40-007 Katowice, Poland; justyna.lipka@us.edu.pl; 2 Department of Psychiatry and Behavioral Sciences, Emory University School of Medicine, Atlanta, GA 30329, USA; jmaple2@emory.edu (J.M.M.-K.); sheila.a.m.rauch@emory.edu (S.A.M.R.); kathryn.black@emory.edu (K.B.); Vmichop@emory.edu (V.M.); brothba@emory.edu (B.O.R.); 3 Emory Healthcare Veterans Program, Emory University School of Medicine, Atlanta, GA 30329, USA; 4 Grady Trauma Project, Grady Memorial Hospital, Atlanta, GA 30303, USA; 5 Atlanta VA Healthcare System, Decatur, GA 30033, USA; 6 Yerkes National Primate Research Center, Emory University, Atlanta, GA 30322, USA

**Keywords:** food addiction, posttraumatic stress disorder (PTSD), sex differences, eating dysregulation, trauma, obesity

## Abstract

Background: Food addiction (FA) is a dysregulated eating pattern characterized by difficulties in controlling the intake of certain foods. There is an overlap in physical and mental health correlates of FA and post-traumatic stress disorder (PTSD). The purpose of this study was to examine sex differences in the rates of positive FA status in individuals with threshold/subthreshold PTSD, and to examine sex differences in the physical and mental health correlates of FA. Methods: Post-9/11 veterans/service members seeking PTSD treatment were recruited. Participants were diagnosed with PTSD via the administration of a clinical interview. FA status was determined using Modified Yale Food Addiction Scale-2, binary sex and body mass index were assessed with demographics questions. Results: Nearly half (43%) of the sample were women. There were no sex differences in the rates of FA, with an overall FA prevalence of 18%. There were no sex differences in FA symptom count in the whole sample (M = 1.63) or those with FA status (M = 6.21). Individuals with FA reported higher frequency of disordered eating, higher severity of PTSD, and depression symptoms. Conclusions: FA should be assessed in tandem with PTSD symptoms, as its prevalence in that sample is higher than in the general population, and it appears to affect both sexes at similar rates.

## 1. Introduction

Food addiction (FA) refers to a constellation of dysregulated eating behaviors analogous to behaviors traditionally associated with substance misuse, such as eating larger amounts or for longer periods than planned, tolerance to certain foods, and persistent and unsuccessful attempts to cut down on eating certain types of food [1,2]. While FA is not a mental health diagnosis, the instrument validated and widely used to measure this construct can be used to generate a cut-off score for identifying individuals at high risk for negative consequences from maladaptive eating patterns (i.e., those with positive FA status vs. those without FA) based on the criteria analogous to those used to identify individuals with alcohol and substance use disorders [1,2]. FA remains a controversial construct: there are arguments that eating, not food itself, has addictive properties (similar to other behavioral addictions), hence making “eating addiction” a more useful construct than “food addiction” [3,4,5]. However, many researchers consider FA a useful construct in identifying individuals at risk for obesity and negative health outcomes related to maladaptive eating patterns, and its clinical utility is increasingly recognized [6]. Positive FA status has been linked to obesity [7] and the presence of eating disorders [8]; it is more prevalent than the DSM-5 eating disorders. In non-clinical community samples, 5–10% of participants met the criteria for a positive FA status [7], with higher prevalence among those with obesity, ranging from 15% to 25% [9,10].

Sex differences in rates of FA have not been studied extensively, and the findings are inconclusive. A meta-analysis of 24 studies found higher prevalence of positive FA status in women [11]. However, most of these studies used samples that had primarily female or female-only representation. One recent study in a primarily female sample found no sex differences in FA prevalence [12], and another found that after controlling for anxiety and perceived stress, sex differences were no longer significant [13]. The National Institutes of Health (NIH) have emphasized the importance of incorporating sex as a biological variable in research in order to identify meaningful sex differences in biopsychological phenomena and in an effort to develop most personalized treatments [14,15]. Given that only a handful of studies examined sex differences in FA and that the samples in most studies to date were skewed toward women, it is important to investigate FA status in samples with equal representation of men and women. Additionally, it appears that stress may be an important factor in accounting for sex differences in FA.

Post-traumatic stress disorder (PTSD) is a heterogenous stress-related psychiatric disorder associated with trauma exposure [16,17]. PTSD is diagnosed after an exposure to a traumatic event (criterion A) [14]. PTSD symptoms involve intrusive memories of the traumatic event (criterion B), avoidance of reminders of trauma (criterion C), negative alterations in cognitions and mood (criterion D), and alterations in arousal and reactivity (criterion E) [17]. Women are more than twice as likely to develop PTSD following trauma exposure [18], including among service members [19]. They also have been shown to experience greater severity of PTSD symptoms compared to men [20].

There is a documented overlap between PTSD symptoms and disordered eating behaviors, particularly loss of control overeating and compensatory behaviors (i.e., vomiting, using laxatives or diuretics, and excessive exercise to compensate for calories consumed) [21,22,23]. Rates of disordered eating for individuals with threshold and subthreshold PTSD are higher in women than in men, similarly to the general population [23]. PTSD is also highly comorbid with alcohol use disorders (AUD), with higher estimates for men compared to women [16]. As FA represents eating behaviors that may encompass disordered eating (particularly loss of control over eating) but also includes behaviors associated with substance use disorders (such as difficulties with quitting certain high-calorie types of food), the rates of positive FA status may be higher in individuals with PTSD than in the general population. Few studies to date examined the link between PTSD and FA, and even fewer included men in their samples; those studies that included men were conducted in veteran samples. One study in primarily male older trauma-exposed veterans found that a lifetime diagnosis of PTSD (assessed using self-report measures) was positively associated with the number of current FA symptoms [24]. In that sample, 2.3% of participants met criteria for positive FA status, with 1.9% of men and 7.3% of women. In a primarily male (89%) sample of older veterans with overweight or obesity seeking weight management treatment, 10% of the sample met criteria for positive FA status [25]. In that sample, FA was also significantly correlated with self-administered PTSD screening. The Nurses’ Health Study II found that the lifetime presence of at least one PTSD symptom was associated with current FA in women [26]. They also found that the severity of PTSD increased the likelihood of having a positive FA status [26]. Other studies in women also found a relationship between childhood trauma and FA [27] as well as current PTSD symptoms and FA [28]. All these studies [26,27,28] used self-report survey measures (as opposed to clinical interviews) to assess PTSD symptoms presence and had female-only samples. Therefore, it is important to examine the rates of positive FA status in men and women diagnosed with PTSD to better characterize their clinical needs and physical health.

There is considerable overlap in certain physical and mental health correlates of FA and PTSD. FA has been found to be associated with BMI in samples with a wide range of body mass and in samples with obesity [7,29]. PTSD has been associated with increased BMI as well as with metabolic complications associated with obesity [30,31]. In fact, it has been proposed that the changes in the sympathetic nervous system and the neuroendocrine system that occur as a result of impaired fear response in PTSD represent a metabolic disorder [32]. PTSD symptoms include re-experiencing unwanted memories of the trauma, difficulties in controlling emotional reactions in response to trauma reminders, and avoidance of external and internal reminders of the trauma [17], leading individuals who experience those symptoms to develop various maladaptive strategies to manage those symptoms. Among them, PTSD has been associated with disordered eating, particularly loss of control over (LOC) eating and compensatory behaviors [21,22,23], similarly to FA [7,33,34,35,36]. In both PTSD and FA, disordered eating research focused primarily on women, and there are fewer studies examining those behaviors in men. PTSD is highly comorbid with depression, with nearly half of individuals with PTSD reporting major depressive disorder [16]. FA has also been found to be associated with depression (for a list of 21 studies, see [33]). Finally, PTSD is highly comorbid with alcohol use disorder (AUD) [37], with estimates of 28% in women, and 52% for men [16]. Interestingly, alcohol use disorder (AUD) has been unrelated to FA in most studies [1,35,38,39,40]. Some have suggested that FA may act as a “replacement” of another addiction [7,41], and there is some evidence to suggest that the relationship between FA and AUD is moderated by third variables [42].

### Study Aims

The purpose of this study was to characterize FA in a sample of individuals with threshold and subthreshold PTSD. We examined sex differences in rates and the following correlates of FA in that sample: body mass index (BMI), frequency of loss of control over (LOC) eating, frequency of compensatory behaviors, severity of PTSD symptoms, severity of depression symptoms, suspected alcohol use disorder (AUD) severity, and frequency of alcohol use. Based on past research, we hypothesized that (1) women would have higher prevalence of positive FA status, compared to men; (2) individuals with positive FA status would have higher BMI than those without FA, regardless of sex; (3) there would be a significant interaction between FA status and sex, such that women with FA would report significantly higher frequency of LOC eating and compensatory behaviors compared to men and to women without FA; (4) there would be a significant interaction between FA status and sex, such that women with FA would report significantly higher PTSD symptom severity compared to men and women without FA; (5) individuals with positive FA status would report significantly higher depression severity, regardless of sex; (6) there would be no significant differences between individuals with and without FA in alcohol use.

## 2. Materials and Methods

### 2.1. Participants and Procedure

All participants were post-9/11 veterans or military service members seeking treatment for PTSD symptoms. The sample was a “convenience” sample of individuals who contacted a specialty treatment program at a medical center, responding to various forms of advertising (e.g., social media, presentations at veteran events, radio spots) or a referral (e.g., from another mental health professional). Patients completed a comprehensive intake evaluation including a structured diagnostic interview for PTSD (Clinician Administered PTSD Scale for DSM-5 [CAPS-5]) [43,44] and a battery of self-report questionnaires to assess symptoms of FA, PTSD, and depression severity, and other health behaviors of interest (LOC eating, compensatory behaviors, alcohol use frequency). Assessors were all either licensed clinical psychologists, or masters or doctoral level trainees under the supervision of a licensed clinical psychologist. The evaluations were conducted either in-person or over the phone, depending on the location of participants. All participants provided informed consent for inclusion before they participated in the study. The protocol was approved by the Institutional Review Board of Emory University School of Medicine.

Inclusion criteria were service members or veterans who served at least one day post 9/11 with current PTSD or subthreshold PTSD (Other Specified Trauma and Stressor-Related Disorder). All participants with subthreshold PTSD met DSM-5 PTSD criteria A (lifetime exposure to a traumatic event) and B (re-experiencing symptoms related to the traumatic event as evidenced by either intrusive memories of the event, nightmares of the event, or emotional and/or physical reactivity in response to specific reminders of the traumatic event). PTSD is a heterogenous disorder and including PTSD A and B criteria in the selection of participants was aimed at reducing further heterogeneity in presentation and to ensure that the Other Specified Trauma and Stressor-Related Disorder (referred to as subthreshold PTSD from now on) is analogous to a threshold PTSD diagnosis.

### 2.2. Measures

#### 2.2.1. Predictor Variables

##### Food Addiction (FA)

The presence of a positive FA status was determined using the Modified Yale Food Addiction Scale—Version 2.0 (mYFAS-2) [2]. The mYFAS-2 operationalizes behavioral indicators of FA based on the DSM-5 criteria for substance use disorders (SUD). The measure consists of 13 self-report items that assess 11 SUD criteria when the substance is certain highly palatable foods, plus clinically significant impairment and/or distress. The measure has two scoring methods. First, the measure is scored to assess a “diagnostic” threshold, which is met if an individual endorses two or more symptoms plus impairment or distress. That method was used to generate the FA status in our sample. Second, there is a continuous scoring method that summarizes how many of the 11 SUD criteria an individual endorsed with respect to consuming highly palatable foods. For individuals who meet the “diagnostic” threshold for FA, the continuous symptoms count method can also be used to specify severity thresholds, with the following categories: 2–3 symptoms = mild, 4–5 = moderate, and 6 or more symptoms = severe. Internal consistency of this measure in our sample was α = 0.90.

##### Sex

Participants reported their sex in a standard demographics questionnaire. The choices available were binary (0 = female, 1 = male). Items regarding participants’ sex assigned at birth and gender identity were not included in this data collection and thus are not reported.

#### 2.2.2. Proposed Correlates

##### Body Mass Index (BMI)

BMI was assessed using participants’ self-reported height and weight. While self-reported BMI tends to be lower compared to the measured BMI, the correlations of self-reported and measured BMI values are very high (Pearson’s *r* correlations above 0.90), and they are equally related to biomarkers of obesity [45]. BMI was calculated using a standard equation [(lbs × 703)/inches squared]. Based on the BMI, weight status was coded as 1 = normal weight (18.5 < BMI < 25), 2 = overweight (25 < BMI < 30), 3 = obese (BMI > 30), 4 = underweight (BMI < 18.5); the cut-offs used were those recommended by the World Health Organization [46].

##### Loss of Control (LOC) Overeating and Compensatory Behaviors

Questions about the frequency of LOC eating and compensatory behaviors (vomiting, laxative/diuretic use, intense exercise) in the past year were administered in addition to the mYFAS-2. Participants were asked to answer the following two items: “I felt loss of control over eating or like I could not stop eating once I started” and “After eating, I did one of the following things to influence my shape and/or weight: made myself vomit, took laxatives, took diuretics, exercised excessively” on a 0–5 Likert scale (0—Never; 1—Less than monthly; 2—Once/month; 3—2 to 3 times/month; 4—Once/week; 5—2 to 3 times/week). These items were also coded dichotomously to identify those who had not endorsed any LOC (LOC in the past year coded as 0) or compensatory behaviors (compensatory behaviors in the past year coded as 0) and those who endorsed any instances of these behaviors (LOC in the past year coded as 1 and compensatory behaviors in the past year coded as 1).

##### PTSD and Depression Symptom Severity

The severity of PTSD symptoms was assessed using the PTSD Symptom Checklist for DSM-5 (PCL-5) [47]. The PCL is a 20-item self-report questionnaire assessing each PTSD symptom in the past month. Participants answer using a 5-point Likert scale (0—Not at all; 1—A little bit; 2—Moderately; 3—Quite a bit; 4—Extremely), with higher score indicating greater symptom severity. The PCL-5 has excellent psychometric properties [47]. Internal consistency of the measure in our sample was α = 0.93.

Depression severity was measured using the Patient Health Questionnaire-9 (PHQ-9) [48]. The PHQ-9 is a nine-item self-report measure on which participants rate their depressive symptoms in the past two weeks on a 4-point Likert scale (0—Not at all; 1—Several days; 2—More than half the days; 3—Nearly every day). Several studies support excellent psychometrics of the PHQ-9 [49,50]. Internal consistency in the current sample was α = 0.82.

##### Alcohol Use Severity and Frequency

Alcohol use severity was measured using the Alcohol Use Disorder Identification Test-Consumption (AUDIT-C) [51]. AUDIT-C is a brief 3-question scale that reliably identified individuals at risk for alcohol use disorders (AUD), including the frequency and amount of alcohol consumed. It is scored on a 0–12 scale, and a score of 3 in women and 4 in men indicates a possible AUD [51,52]. AUDIT-C has excellent psychometrics. In the current sample, the internal consistency was α = 0.82. To assess the frequency of alcohol use, participants identified the number of days in the past 14 on which they had an alcoholic beverage.

#### 2.2.3. Demographics and Covariates

##### Demographics

All participants completed a brief demographics questionnaire in which they identified their sex, age (in years), race, ethnicity, weight, and height. Race was assessed using seven options: White/Caucasian, Black/African American, Asian, Native Hawaiian or Other Pacific Islander, American Indian/Alaskan Native, Other/Biracial/Multiracial, and Don’t Know). Ethnicity was coded as 0 = non-Hispanic/Latinx, 1 = Hispanic/Latinx.

##### Covariates

Given that the current sample consisted of post-9/11 veterans who met criteria for either threshold and subthreshold PTSD diagnosis, PTSD status was coded (1 = threshold, 0 = subthreshold) and used as a covariate in all models.

The number of lifetime traumatic events was included in the initial descriptive statistics to be considered as a possible covariate were sex differences found. The Life Events Checklist [53] is a measure of exposure to potentially traumatic events. The LEC exhibits strong temporal stability and convergent validity [53]. Participants’ total trauma exposure score represents a sum score of each trauma type that they reported directly experiencing.

### 2.3. Data Analyses

To test study hypotheses, we conducted a set of 2 × 2 ANOVAs, with sex (female, male) and FA status (positive, negative) as between-group factors; the outcome variables were BMI, dysregulated eating variables (LOC eating and compensatory behaviors), PTSD severity, depression severity, AUD severity, and alcohol use frequency. We identified covariates as the demographic variables that were significantly different between women and men using χ^2^ tests for categorical variables and independent samples *t*-tests for continuous variables. We conducted bivariate Pearson’s correlations to identify significant relationships between FA severity (continuous scoring) and the covariates of interest. All analyses were conducted using SPSS Version 27.

## 3. Results

### 3.1. Participant Chracterstics

The current sample consisted of 214 post-9/11 veterans (43% women, mean age: 41 years; see Table 1). The participants in the current sample were majority White (62%), followed by Black (29%), and majority non-Hispanic/Latinx (85%). The majority of participants had other than the normal weight, with 36% reporting overweight and 42% reporting obesity; the mean BMI was 29.74 (see Table 1). Men were significantly older than women (*p* = 0.037); the sample consisted of significantly more Black women (45%) than Black men (17%) and significantly more White men (76%) than White women (44%) (*p* < 0.001). The majority of participants met criteria for threshold (vs. subthreshold) PTSD diagnosis (78.5%), with significantly more women (91%) compared to men (69%) meeting the criteria for threshold vs. subthreshold PTSD (*p* < 0.001). Therefore, age, race, and PTSD status were used as covariates in all ANOVA models.

### 3.2. Rates of Food Addiction

In the current sample, 18% (*n* = 38) of participants met criteria for a positive FA status. There were no statistically significant sex differences in the prevalence of FA (χ^2^ = 0.09, *p* = 0.761.

There were no statistically significant sex differences in the number of FA symptoms reported in the entire sample (*p* = 0.241) or among individuals with a positive FA status (*p* = 0.121; see Table 1 for both). The majority (61%) of participants with a positive FA status endorsed symptoms in the severe range. There were no statistically significant sex differences between the severity categories (χ^2^ = 1.84, *p* = 0.398), with 71% of women and 52% of men reporting severe FA symptoms in the past year (see Table 1).

### 3.3. Body Mass Index (BMI)

**Figure 1:**
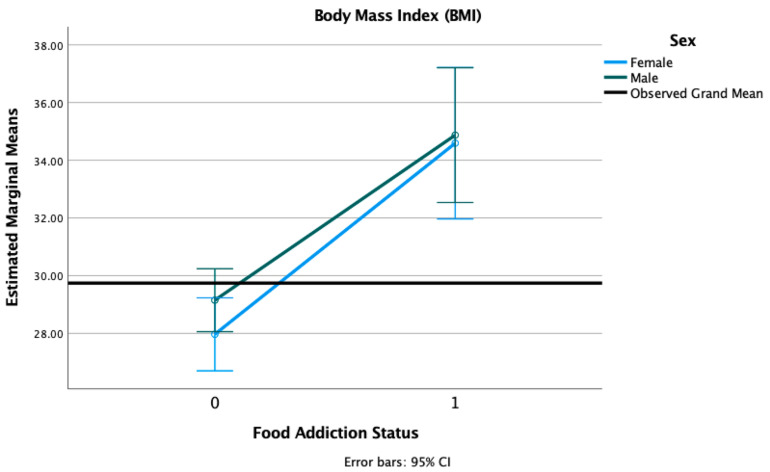
Estimated marginal means of body mass index (BMI) for a 2 × 2 factorial ANOVA with sex (female, male) and FA status (1 = positive, 0 = negative, on the *X*-axis) adjusted for age, race, and PTSD status (threshold vs. subthreshold).

We conducted 2 × 2 factorial ANOVA with sex (female, male) and FA status (positive, negative) adjusted for age, race, and PTSD status. We found a main effect of FA diagnosis (F = 39.62, *p* < 0.001), such that individuals with FA had significantly higher BMI (M = 34.69, SD = 7.23) compared to those without FA (M = 28.68, SD = 5.13). There was no main effect of sex (F = 0.53, *p* = 0.468) nor a sex × FA interaction (F = 0.21, *p* = 0.647) (see Figure 1).

### 3.4. LOC Eating and Compensatory Behaviors Frequency

**Figure 2:**
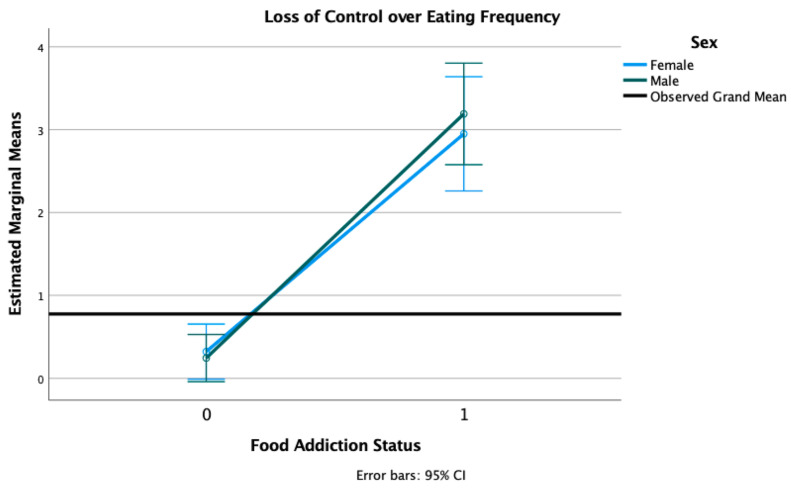
Estimated marginal means of loss of control over eating frequency for a 2 × 2 factorial ANOVA with sex (female, male) and FA status (1 = positive, 0 = negative, on the *X*-axis) adjusted for age, race, and PTSD status (threshold vs. subthreshold).

**Figure 3:**
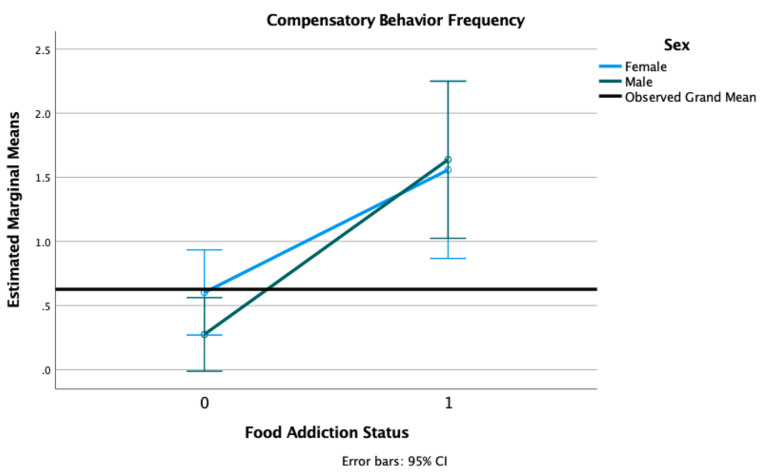
Estimated marginal means of compensatory behaviors frequency for a 2 × 2 factorial ANOVA with sex (female, male) and FA status (1 = positive, 0 = negative, on the *X*-axis) adjusted for age, race, and PTSD status (threshold vs. subthreshold).

In the current sample, 22% of participants reported at least one episode of LOC and 21% reported at least one episode of compensatory behaviors in the past year (see Table 1). There were no sex differences in the prevalence of LOC eating or compensatory behaviors. We conducted 2 × 2 factorial ANOVAs with sex (female, male) and FA status (positive, negative), adjusted for age, race, and PTSD status. We found a main effect of FA status on LOC eating frequency (F = 117.32, *p* < 0.001) and compensatory behaviors frequency (F = 53.96, *p* < 0.001), with individuals with FA reporting significantly more LOC episodes (M = 3.08, SD = 2.78) than those without FA (M = 0.28, SD = 0.89) and significantly compensatory behaviors (M = 2.94, SD = 2.69) than those without FA (M = 0.30, SD = 2.69). There was no main effect of sex (F = 0.09, *p* = 0.757) or a sex × FA interaction (F = 0.38, *p* = 0.537) present for LOC eating frequency (see Figure 2). There was no main effect of sex (F = 0.21, *p* = 0.641) nor a sex × FA interaction (F = 0.62, *p* = 0.431) present for compensatory behaviors frequency (see Figure 3).

### 3.5. PTSD and Depression Symptom Severity

**Figure 4:**
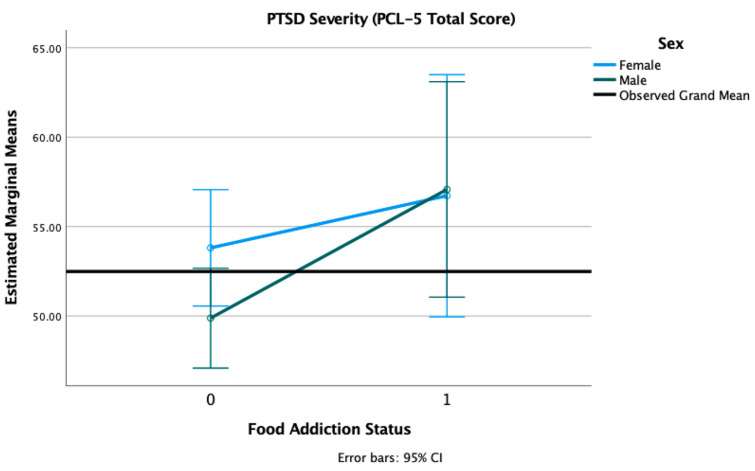
Estimated marginal means of PTSD severity for a 2 × 2 factorial ANOVA with sex (female, male) and FA status (1 = positive, 0 = negative, on the *X*-axis) adjusted for age, race, and PTSD status (threshold vs. subthreshold). Note: PCL-5 = PTSD Symptom Checklist for DSM-5.

We conducted 2 × 2 factorial ANOVAs with sex (female, male) and FA status (positive, negative) adjusted for age, race, and PTSD status, with PTSD severity and depression severity as outcome variables. We found a main effect of FA on PTSD symptoms severity (F = 4.00, *p* = 0.047), such that individuals with a positive FA status reported higher PTSD symptoms severity (M = 57.68, SD = 10.53) than those without FA (M = 51.37, SD = 15.30). There was no main effect of sex (F = 0.48, *p* = 0.490) nor a sex × FA status interaction (F = 0.72, *p* = 0.398) (see Figure 4).

**Figure 5:**
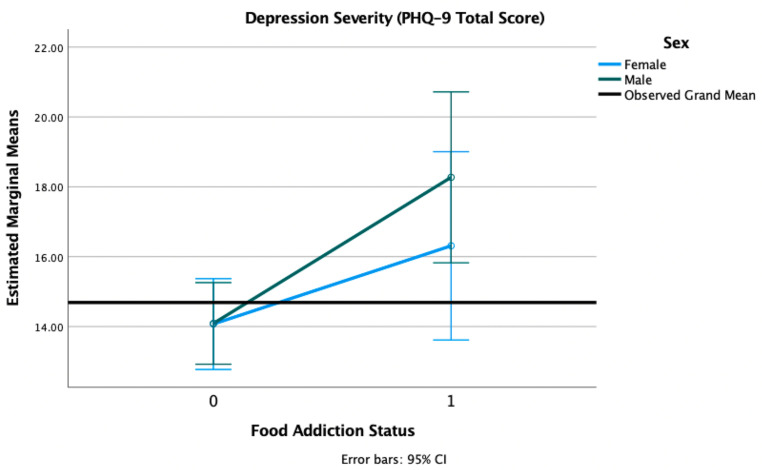
Estimated marginal means of depression severity for a 2 × 2 factorial ANOVA with sex (female, male) and FA status (1 = positive, 0 = negative, on the *X*-axis) adjusted for age, race, and PTSD status (threshold vs. subthreshold). Note: PHQ-9 = Patient Health Questionnaire.

We found a main effect of FA on depression symptom severity (F = 9.99, *p* = 0.002), such that individuals with a positive FA status reported higher depression severity (M = 17.57, SD = 5.23) than those without FA (M = 14.04, SD = 5.53). There was no main effect of sex (F = 0.899, *p* = 0.344) nor a sex × FA status interaction (F = 0.910, *p* = 0.341) (see Figure 5).

### 3.6. Alcohol Use Severity and Frequency

**Figure 6:**
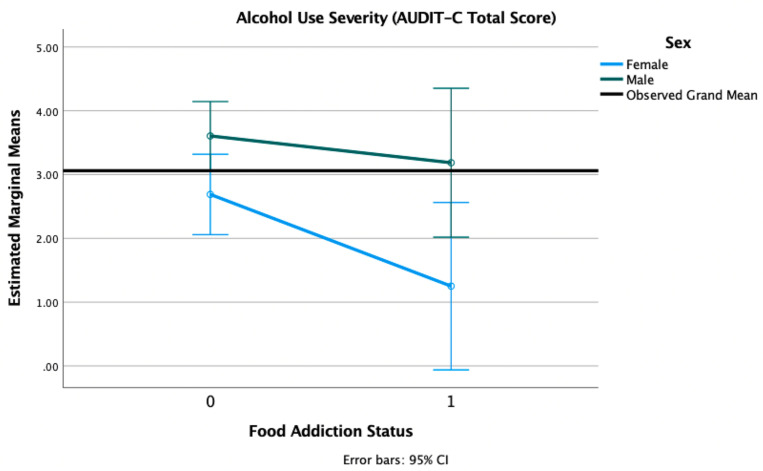
Estimated marginal means of alcohol use severity for a 2 × 2 factorial ANOVA with sex (female, male) and FA status (1 = positive, 0 = negative, on the *X*-axis) adjusted for age, race, and PTSD status (threshold vs. subthreshold). Note: AUDIT-C = Alcohol Use Disorder Identification Test-Consumption.

**Figure 7:**
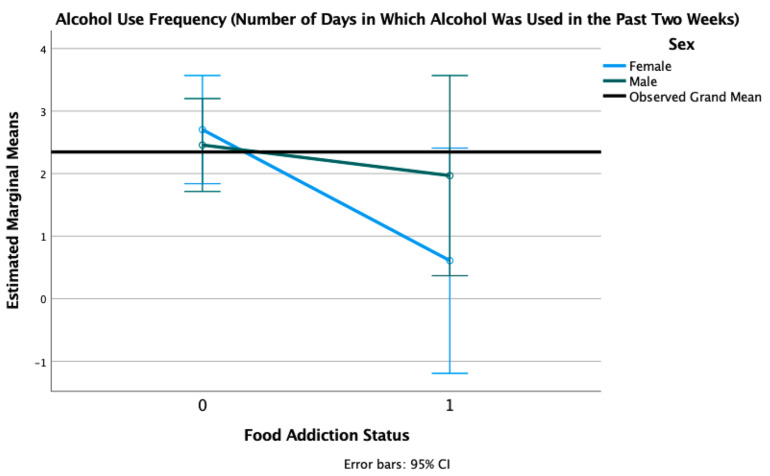
Estimated marginal means of alcohol use frequency for a 2 × 2 factorial ANOVA with sex (female, male) and FA status (1 = positive, 0 = negative, on the *X*-axis) adjusted for age, race, and PTSD status (threshold vs. subthreshold).

We conducted 2 × 2 factorial ANOVAs with sex (female, male) and FA status (positive, negative) adjusted for age, race, and PTSD status, with alcohol use disorder (AUD) severity measured by the AUDIT-C and alcohol use frequency (number of days when alcohol was used in the past 14) as outcome variables. There was an expected main effect of sex (F = 8.09, *p* = 0.005) on the AUD severity (as the cut-off is higher for men compared to women), such that men reported significantly higher AUD severity (M = 3.210, SD = 3.068) than women (M = 2.516, SD = 2.208). There was only a trend level main effect of FA on AUD severity (F = 3.60, *p* = 0.059). There was no sex × FA interaction (F = 1.08, *p* = 0.300) (see Figure 6). There was no main effect of sex (F = 0.66, *p* = 0.419), only a trend-level effect of FA (F = 3.70, *p* = 0.056), and no sex × FA interaction (F = 1.43, *p* = 0.234) for the number of days in which alcohol was used in the past two weeks. In both of these models, the trend-level main effects of FA indicated lower AUD severity and fewer days of alcohol use in those with positive FA status, compared to those without FA (see Figure 7).

## 4. Discussion

The purpose of the present study was to characterize food addiction (FA) in a sample of women and men with threshold and subthreshold PTSD, with particular emphasis on investigating sex differences in the prevalence and correlates of a positive FA status in that sample. Contrary to our hypothesis (1), there were no sex differences in the prevalence of FA in that sample, with the overall prevalence of 18%. As predicted in hypothesis (2), individuals with a positive FA status had significantly higher BMI than those without FA, regardless of sex. Hypothesis (3) was partially supported in that individuals with a positive FA status reported engaging in loss of control (LOC) eating and compensatory behaviors significantly more frequently than individuals without FA, but this effect was the same for women and men, as we found no interaction between FA status and sex. Similarly, hypotheses (4) and (5) were partially supported in that individuals with a positive FA status reported significantly higher severity of PTSD and depression symptoms, but these effects did not vary between women and men. Finally, consistent with hypothesis (6), there were no significant differences between individuals with and without FA in alcohol use disorder (AUD) severity and alcohol use frequency; however, we found trend-level effects of FA, such that individuals with a positive FA status trended to report lower AUD severity and lower frequency of alcohol use.

The findings of the current study suggest that PTSD symptoms overlap with a positive FA status at relatively high rates compared to the general population, and that it is important to assess FA in both men and women with PTSD. Traditionally, eating disorders have been associated with and studied in women while often being overlooked in men [54]. In the current sample, 18% of participants met criteria for FA, with comparable rates in men (17%) and women (19%). A recent meta-analysis indicated an overall prevalence of FA across samples at 16%, with a caveat that the majority of participants (over 70%) in the reviewed studies were women [33]. One study in a highly traumatized sample of women found comparable rates of FA (18%) [28]. This is the first study to use approximately equal samples of women and men to assess sex differences in FA status.

While we found no sex differences in the proposed correlates of FA—BMI, LOC eating, compensatory behaviors, PTSD severity, and depression severity—we did find that individuals with a positive FA status significantly differed on each of these correlates from individuals without FA. For individuals with FA, BMI on average fell in the obese range, while for those without FA, it was on average in the overweight range. Since PTSD has been found to be associated with obesity [30,31,32], our findings suggest that eating patterns present in FA may be one behavioral pathway to obesity and adverse health outcomes in individuals with PTSD. Therefore, it may be particularly important to study individuals who present with both PTSD and FA, as they may be at highest risk for experiencing adverse health outcomes. However, that is an empirical question for further investigation. Of note, the rate of obesity in our sample was on par with the most recent U.S. national estimate among women (approximately 40%), but higher than the national estimate for men (35% national average vs. nearly 44% in our sample) [55]. Thus, it is possible that there are sex differences in the mechanisms that affect development of obesity in men and women following exposure to traumatic events. A positive association between FA and disordered eating behaviors, particularly LOC eating and compensatory behaviors, is also consistent with past findings [33,34,36,56,57], but it was unexpected to find no sex differences in LOC eating and compensatory behaviors. In the general population, disordered eating behaviors are reported at higher rates by women [17]. However, some studies have suggested that there may be more similarities than differences in FA between men and women. One study in a large sample of French college students found that after controlling for anxiety and perceived stress, the differences in FA severity between women and men were not significant [13]. The sample in the current study consisted of individuals with a diagnosed trauma and stress-related disorder, and all analyses were adjusted for the type of diagnosis. It is possible that men are more susceptible to using food as a coping and emotion regulation strategy following stressful or traumatic experiences than previously thought. In our sample, we found that at the statistical trend level, individuals with a positive FA status (regardless of sex) tended to have lower AUD severity and alcohol use frequency. This may serve as potential evidence for the substitute addiction hypothesis, wherein certain individuals with biological predispositions toward developing an addiction turn to food as opposed to alcohol or drugs [7,58]. It will be important to examine mechanisms of development and maintenance of FA in individuals exposed to trauma to better understand what biopsychological factors increase the risk of FA following trauma. It may be of particular importance now, in the midst and the aftermath of the global COVID-19 pandemic, in which a large number of people has been exposed to prolonged stress and traumatic events [59,60].

It is consistent with past research that individuals with FA reported higher severity of PTSD and depression symptoms [33]. However, it was unexpected that there were no sex differences in the severity of PTSD symptoms. Women have been shown to be twice as likely to develop PTSD following trauma exposure [18], including among service members [19], and they tend to exhibit greater PTSD symptoms [21]. In the current study, the Student’s *t*-test to examine sex-based group differences revealed that women indeed reported higher severity of PTSD symptoms. However, in the full model that accounted for the type of diagnosis, age, and race, this effect was no longer significant. As significantly more women met criteria for PTSD vs. Other Specified Trauma and Stressor disorder, compared to men, it is possible that the type of diagnosis had accounted for potential sex differences in severity of PTSD.

As expected, we found no differences in AUD severity and alcohol use frequency between FA and non-FA groups. This is consistent with past research, indicating that FA is not associated with alcohol use disorders (for a review, see [7,29]). It has been suggested that the relationship between AUD and FA may be more complex and moderated by third variables [41,42]. As mentioned earlier in this section, FA may also serve as a “replacement addiction”, making people with FA less likely to meet criteria for other addictions. Interestingly, the average frequency of alcohol use in our sample was relatively low at two days out of the past two weeks. Among people with the positive AUD screen, the average number of days of alcohol use was five in the past two weeks. Given that nearly half of our sample (44%) met or exceeded the screening cut-off for a possible alcohol use disorder, it is possible that the majority of participants in our sample tended to be heavy drinkers on days on which they used alcohol.

Overall, the results of the current study are consistent with past research on the correlates of FA, suggesting that mYFAS may be successfully used with individuals with PTSD. These results also suggest that there may be a subtype of individuals who are more susceptible to developing FA following trauma than others. These individuals also report higher severity of mental health symptoms and may be at higher risk for developing adverse health outcomes secondary to obesity.

One of the weaknesses of the current study is the lack of assessment of gender identity. Participants were asked to report their biological sex but were not asked to identify their gender identity. Therefore, in future research, it will be important to include questions regarding gender identity and its potential effects on FA. As the data for this study were collected in a clinical sample of treatment-seeking individuals, the study design lacks a non-PTSD control group. Future investigation would benefit from a matched control group to further understand the role of FA in PTSD. Another potential weakness is that this study used the validated brief version of the YFAS. This may have introduced bias in the rates of FA, as past studies found slightly lower rates of a positive FA status when it was measured by the mYFAS compared to the full YFAS [61]. On the flipside, mYFAS is faster to administer and was a better fit for a clinical setting in which this study was conducted. To reduce the participant burden in a primarily clinical setting, we were unable to include another validated measure of eating behavior. Although we included questions about loss of control over eating and compensatory behaviors, we did not assess restraint over eating or eating behaviors consistent with anorexia nervosa. This is a weakness, as FA has been linked to anorexia nervosa in an adolescent sample [62]. Another weakness of the present study was the use of self-report as opposed to measured BMI. However, while self-reported BMI tends to be lower when compared to measured BMI, the correlations between them are very high, and they are equally related to biomarkers of obesity [45]. Finally, all measures used in the study were self-report measures, as even clinical interviews rely heavily on participants’ report. Therefore, the presence of potential response biases should be acknowledged.

One of the strengths of the current study is the sample has a roughly half and half composition of women and men, allowing for sufficiently powered examination of sex differences. While we did not purposely oversample women, heavy involvement of women in post-9/11 operations (including in combat), greater attention to the effects of military sexual trauma in recent years, and greater risk of developing PTSD following traumatic events for women likely contributed the relative overrepresentation (compared to overall representation of women in active duty and the reserves) in our sample [18,19,63]. Another strength is the use of a clinical sample whose diagnosis was ascertained by a gold standard clinical interview administered by highly trained clinicians. Finally, examining sex differences addresses an important area of research and is consistent with the NIH’s calls for developing more precise and personalized interventions.

Future research should focus on the mechanisms of the relationships between trauma exposure and FA in individuals with and without PTSD. As individuals with PTSD have higher rates of adverse health outcomes compared to the general population, FA symptoms may be an effective therapeutic target that addresses distress related to eating dysregulation as well as metabolic sequelae. Thus, it is important to identify potential mechanisms of action between FA and metabolic health components in that sample, and to identify whether there is a distinct PTSD + FA phenotype to provide the most effective and personalized interventions.

## 5. Conclusions

The findings of this study highlight the importance of assessing symptoms of FA in individuals who present to treatment with PTSD diagnosis, and the importance of paying attention to these symptoms in men. Individuals with PTSD are at increased risk for adverse metabolic health outcomes; therefore, monitoring FA symptoms in patients with PTSD and intervening when indicated may help ameliorate those negative health consequences. These findings also suggest that the brief mYFAS is an effective tool for assessing eating dysregulation in busy clinical settings. It appears that in individuals with a diagnosis of a trauma or stress-related disorder, sex differences in dysregulated eating are much less prominent than in the general population; therefore, it may be a population of special interest for developing more effective and personalized interventions for both PTSD and eating dysregulation.

## Figures and Tables

**Table 1:** Participant characteristics.

		%/M (SD)				
		Whole Sample (*n* = 214)	Women (*n* = 91)	Men (*n* = 123)	χ^2^/t	*p*
Age		40.88 (9.51)	39.30 (9.78)	42.04 (9.18)	2.09	0.037 *
Race					28.74	<0.001 **
	African American or Black	28.97	45.06	17.07		
	Caucasian or White	62.62	43.96	76.42		
	Naive Hawaiian or Other Pacific Islander	0.47	0.00	0.81		
	Asian	0.94	1.10	0.81		
	American Indian or Alaska Native	1.40	2.20	0.81		
	Biracial/Multiracial	2.80	5.50	0.81		
	Don’t know	2.80	2.20	3.25		
Ethnicity					4.39	0.111
	Non-Hispanic or Latino	85.05	86.81	83.74		
	Hispanic or Latino	8.41	9.89	7.32		
	Missing	6.54	3.30	8.94		
PTSD Status					15.14	<0.001 **
	Threshold	78.5%	91%	69%		
	Subthreshold	21.5%	9%	31%		
BMI		29.74 (6.00)	28.86 (5.86)	30.40 (6.05)	1.87	0.064
Weight status					4.41	0.220
	Normal weight	20.09	24.18	17.07		
	Overweight	35.51	32.97	37.40		
	Obese	42.52	40.66	43.90		
	Underweight	0.94	2.20	0.00		
	Missing	0.94	0.00	1.63		
Food Addiction Diagnosis					0.09	0.761
	Positive	17.76	18.68	17.07		
	Negative	82.24	81.32	82.93		
Food Addiction Symptom Count	Whole Sample	1.63 (2.54)	1.87 (2.75)	1.46 (2.37)	1.18	0.241
Food Addiction Symptom Count	Positive FA Status ^+^	6.21 (2.39)	6.88 (2.20)	5.67 (2.46)	1.59	0.121
Food Addiction Severity ^+^					1.84	0.398
	Mild	13.16	5.88	19.05		
	Moderate	26.32	23.53	28.57		
	Severe	60.53	70.59	52.38		
LOC eating					0.28	0.598
	Present	22.4%	24.2%	21.1%		
	Absent	77.6%	75.8%	78.9%		
Compensatory Behaviors					1.56	0.211
	Present	21.2%	25.3%	18.2%		
	Absent	78.8%	74.7%	81.8%		
	Missing	0.9%	0%	1.6%		
LOC Eating Frequency		0.78 (1.77)	0.82 (1.79)	0.74 (1.77)	−0.34	0.732
Compensatory Behaviors Frequency		0.63 (1.49)	0.80 (1.67)	0.50 (1.34)	−1.48	0.139
PCL		52.49 (14.75)	55.42 (14.31)	50.33 (14.75)	−2.53	0.012 *
LEC		6.88 (2.63)	6.69 (2.63)	7.02 (2.63)	0.89	0.374
PHQ		14.69 (5.63)	14.86 (5.73)	14.55 (5.58)	−0.37	0.710
Possible Alcohol Use Disorder (AUDIT-C)					0.08	0.775
	Positive	43.93	45.05	43.09		
	Negative	56.07	54.95	56.91		
Days Used Alcohol		2.35 (3.72)	2.35 (3.79)	2.34 (3.68)	−0.01	0.989

^+^ Values reported only for participants with a positive FA status (*n* = 38). The sample sizes are as follows: Women *n* = 17; Men *n* = 21. Note. Continuous variables are presented as mean (standard deviation), and categorical variables are presented as percentage. PTSD = Post-traumatic Stress Disorder; BMI = Body Mass Index; LOC eating = loss of control over eating; PCL = PTSD Symptoms Checklist; LEC = Lifetime Events Checklist; PHQ-9 = Patient Health Questionnaire; AUDIT-C = Alcohol Use Identification Test-Consumption. Note. ** indicates *p* < 0.01; * indicates *p* < 0.05.

## Data Availability

Data available upon request from the corresponding author.
